# 3D Printing of Elastic Membranes for Fluidic Pumping and Demonstration of Reciprocation Inserts on the Microfluidic Disc

**DOI:** 10.3390/mi10080549

**Published:** 2019-08-19

**Authors:** Maria Bauer, Adrian Bahani, Tracy Ogata, Marc Madou

**Affiliations:** 1 Department for Mechanical and Aerospace Engineering, University of California, Irvine, CA 92697, USA; 2 Department of Biomedical Engineering, University of California, Irvine, CA 92697, USA; 3 School of Engineering and Sciences, Tecnologico de Monterrey, Monterrey 64849, Mexico

**Keywords:** material jetting, CD microfluidics, elastic membrane printing, microfluidics, fluid reciprocation

## Abstract

While 3D printing is increasingly used in most fields of engineering, its utilization for microfluidics has thus far been limited. To demonstrate future applications of 3D printing for microfluidic structures, we investigate the fluidic characteristics of material jetted surfaces. We also demonstrate the manufacture of dual-material microfluidic inserts that feature rigid and elastic elements. The fabricated parts are inserted on a microfluidic CD, enhancing design freedom and prototyping capability of over molded parts. Furthermore, printed elastic membranes are tested for fatigue during elastic-pneumatic pumping and rigid and elastic surfaces are characterized with regards to hydrophilicity and surface topography. Finally, different printed disc inserts are demonstrated for moving liquid towards the center of rotation, the mixing of liquids, and controlling burst events through channels width.

## 1. Introduction

While Compact Disc (CD) microfluidic platforms inherently provide pumping pressure from the center to the rim based on the centrifugal force, the capability of reverting flow back towards the center and radial flow reciprocation are valuable additions in the microfluidic toolbox [1,2,3,4]. Reciprocating flow not only enhances mixing but also improves the lower limit of detection (LOD) of biomarkers [2]. For example, in the case of an assay utilizing a capture probe, repeated flow over a functionalized sensor area can increase the amount of analyte captured in a given amount of time [5] and can therefore yield higher sensitivity, while lowering the limit of detection (LOD). In electrochemical detection, flow provides signal enhancement through enhanced mass transport [6,7]. Furthermore, reverting and repeating flow is especially helpful when working with small sample volumes, where the volume would otherwise not suffice to provide continuous flow throughout the duration of the measurement [8].

In CD microfluidics, liquid is actuated through centrifugal pressure ($P_{c}$), which is calculated based on Equation (1). The faster the disc is spun the higher the pressure on a liquid column in a channel [9]:

(1) $$P_{c}=\frac{1}{2}\rho \omega^{2}\left(r_{A}^{2}-r_{R}^{2}\right)$$

where *ρ* is the density of the fluid, *ω* is the angular velocity (spin speed), and $r_{A}$ and $r_{R}$ are the distance from the rotation center to the advancing and receding fluid front, respectively.

**Figure 1:**
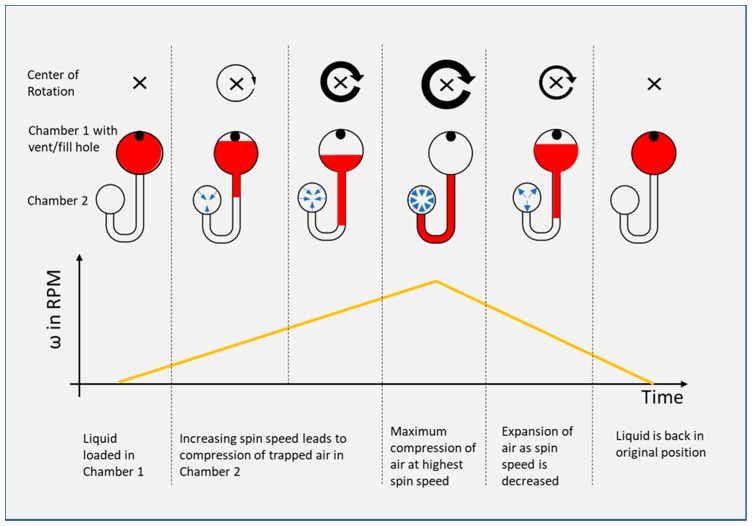
Basic principle of an air compression chamber for (ideal) reciprocation of fluid on a microfluidic disc.

Previous work has demonstrated an un-vented compression chamber, in which the trapped air is compressed as the liquid advances at increasing spin speeds. Subsequent decrease of the angular velocity allows the air to expand and, thence, pushes the liquid back towards the center. The effect is based on Boyle’s law for an ideal gas at constant temperature, which is given in Equation (2).

(2) $$p_{1}V_{1}=p_{2}V_{2}$$

with $p_{1}$ and $V_{1}$ as the pressure and volume of an ideal gas at time 1 and $p_{2}$ and $V_{2}$ as the pressure and volume at time 2. The principle of flow reciprocation through compression and expansion of air was demonstrated by Noorozi et al. [2] and the effect was amplified through addition of an elastic membrane atop of the air compression chamber [3]. Analytical modeling of the elastic membrane is discussed by Aeinevhand et al. [3]. The principle of an air compression chamber for liquid reciprocation on a microfluidic CD is illustrated in Figure 1. Other related reciprocation principles include heating of the unvented air chamber to cause expansion of the enclosed air [10] and electrolysis by integrated electrodes. The generated hydrogen and oxygen gases increase the volume and pump the liquid back to the center [11].

Pneumatic pumping with an elastic membrane increases the efficiency of the reciprocation [3] and allows the reciprocation to take place at lower spin speeds. As a consequence, less expensive motors can be used and the bonding between CD layers endures less stress. However, the assembly process of the microfluidic disc prototypes with elastic membranes is time consuming and the CD assembly process introduced by Aeinevhand et al. includes the manual alignment and adhesion of seven different layers [3].

Multi-material printing has previously been demonstrated to allow for greater flexibility than traditional fabrication while combining functionalities of soft and hard materials. For example Mohammed et al. [12] demonstrated enhanced capabilities of 3D printing by producing facial protheses for ears and noses, where the combination of soft and hard materials allows for modelling of cartilage, bone, and skin. Moreover, MacCurdy et al. demonstrated all-in-one fabrication of prefilled hydraulic systems based on 3D printing of a combination of liquid, rigid, flexible, and support material [13], thus creating fluid filled microfluidics in one printing step. Finally, combinations of printed materials on the micro and macro scale have been extensively reviewed by Molotnikov et al. [14].

Methods allowing for multi material printing include fused deposition modelling (FDM) and material jetting. To create a part with FDM, a thermoplastic filament is heated above its glass transition temperature, deposited on the printing platform, and then re-solidifies. While FDM is a well-known and very affordable approach, part quality suffers for fluidic applications from imperfections in bonding between layers. This is especially the case between layers of different materials [15,16]. Material jetting, which is based on the deposition of droplets from ink jet nozzles, allows for printing multiple materials without gaps between layers.

Here, we therefore present an approach based on material jetting to prototype reciprocation inserts for centrifugal microfluidics in one single step, enabling fast prototyping of microfluidic structures for fluid reciprocation, while producing structures which resemble over-molded mass manufactured parts. Furthermore, we demonstrate these CD microfluidic inserts for reciprocation-based mixing and specifically characterize the surfaces with regard to use for microfluidic applications. The mechanical characterization on 3D printed materials is described elsewhere [17].

## 2. Materials and Methods

An Objet260 Connex3 (Stratasys Ltd., Rehovot, Israel) printer was used for the fabrication of the microfluidic structures in this work. This printer is based on material jetting of UV hardening resin, in which liquid photoactive polymer droplets are expelled from a printer head and sprayed onto a building platform. The deposited droplets are then cured with UV light. After the first layer of the structure is printed, the building platform is lowered, and a second layer is built on top of the first. This process continues until the entire structure is finished. Multiple printheads allow for multi-material prints and the use of droplets of different photoactive polymers allows for the creation of material gradients.

Material jetting allows for two types of surface finishes on the top and side surfaces of a part, i.e., *matte* and *glossy*. The respective finish depends on the presence or absence of support material. Support material is sacrificial material with weaker bonds that can be mechanically (i.e., via a water jet) or chemically (dissolved by submerging the part in a solvent) removed. Print support is essential for parts that otherwise would be suspended and could not stay in place unless they are supported. If a surface is in contact with support material the surface will obtain a matte finish, while support-material-free surfaces result in a glossy finish.

Materials used for parts in the scope of this work were VeroWhitePlus, VeroCyanPlus (both rigid and opaque), and TangoBlackPlus (rubber-like) (all materials obtained from Stratasys Ltd., Rehovot, Israel). Parts were first designed in SolidWorks 2016 (Dassault Systemes, Vélizy-Villacoublay, France) and then exported as an STL file to Objet Studio (Stratasys Ltd., Rehovot, Israel). The mixing efficiency was simulated using SolidWorks 2018 FloXpress (Dassault Systemes, Vélizy-Villacoublay, France).

The sacrificial support material in enclosed microfluidic structures (channels) could not entirely be removed. Therefore, structures were printed that were open on one side and were then sealed with a single sided adhesive (ARCare, Adhesives Research, Glen Rock, PA, USA) and attached onto acrylic holders in order to be mounted on the CD and the testing spin stand. The liquid motion on the spinning platform was visualized through a combination of a trigger, a strobe light, and a high-speed camera, as described elsewhere [7]. To demonstrate mixing, fluid reciprocating CD inserts with different channel geometries were produced. Furthermore, surface characterization inserts with different surface finishes (see Figure 5) were printed with VeroWhitePlus. The nominative depths of all the channels was 1 mm, while the width was varied between 0.1 mm, 0.25 mm, 0.5 mm, 0.75 mm, and 1 mm. After loading of 20 uL dyed deionized water in each loading chamber, the burst of liquid through channels was observed while increasing spin speed in 10 rounds per minute (RPM) steps. Each burst event was repeated 10 times and the respective spin speed averages and standard deviations were calculated.

## 3. Results and Discussion

### 3.1. Design of Dual Material Inserts

**Figure 2:**
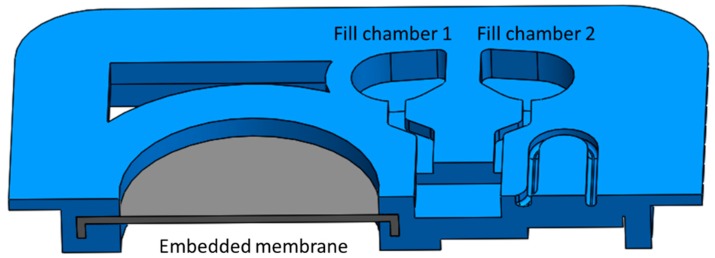
Embedded rubber-like membrane. Embedding the elastic membrane is necessary to avoid delamination.

**Figure 3:**
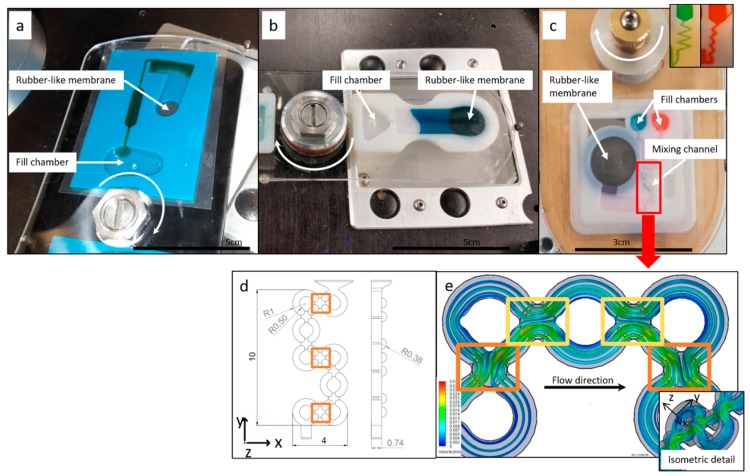
Three different reciprocation inserts are shown mounted on the spin stand. In all cases, liquid is loaded into the fill chamber(s) and then released by overcoming capillary forces in outlet channel (burst valve). (**a**) Reciprocation insert based on compression of air and elastic deformation of rubber-like membrane. (**b**) Straight-channel reciprocation insert to decouple printed micro-balloon performance, used for testing of the rubber-like membrane for possible fatigue. (**c**) Mixing insert allowing for the mixing of two components during reciprocation. (**d**) Major dimensions of the mixing channel [in mm]. (**e**) Water flow path during reverted flow simulation at a 200 µL/min flow rate; the orange marks show locations of increased liquid interchange between merging and separating channels and the yellow marks show locations with less liquid interchange between merging channels (compare over-crossing of green).

To assign different materials to sections of the same print, two parts were designed in SolidWorks 2016 (one for each material) and an assembly was created. Respective materials were then assigned to the different parts of the assembly in Objet Studio (Stratasys Ltd., Rehovot, Israel). Since initial designs made without securing the rubber-like membrane in the rigid part properly led to delamination, in all parts discussed in this work, the membrane was embedded in the rigid material, as shown in the cross-sectional view of the model in Figure 2 (a photograph of the same part is shown in Figure 3c). The optimal thickness of the rubber-like membrane for maximum expansion, while maintaining sufficient stability, was found to be around 200 μm.

### 3.2. Variants of Reciprocation Inserts

In Figure 3 we show the three different types of 3D printed reciprocation inserts fabricated in this work. While all the structures in Figure 3 could be produced through a combination of traditional machining and layered assembly, with one layer being an elastic membrane (as demonstrated by Aeinehvand et al. [3]), the introduced geometries demonstrate applications for 3D printed reciprocation inserts, significantly simplifying assembly and integration on the spinning platform. In all designs, liquid is loaded into a fill chamber and released by increasing the centrifugal pressure over the burst pressure of the capillary burst valves. In Figure 3a we show an insert with an unvented air-chamber with a rubber-like membrane to push the fluid back to the center. Liquid reciprocation was demonstrated with this insert at spin speeds as low as 400 RPM. In Figure 3b we show an insert with a straight-channel, thus only utilizing the elastic deformation of the rubber-like membrane for fluid reciprocation. The behavior of this membrane is further discussed in Section 3.3. While this geometry does not allow the liquid to revert back to its loading position, reciprocation allows for repeated flow over an area of interest close to the elastic membrane position. In Figure 3c (bottom) we present a mixing insert consisting of two loading chambers, a third collecting chamber that can be used for detection, and a *woven* channel (channel detail shown in Figure 3d,e) leading to a compression chamber with an elastic membrane and, finally, a siphon leading from the third chamber to a waste chamber. The woven channel consists of a geometry which repeatedly separates a fluid from a single channel into two channels, which loop around and reunite the fluid paths; thus, splitting and re-merging fluid repeatedly. With this channel geometry, homogeneous mixing was visually observed after 2–3 cycles of reciprocation compared to 5–6 cycles in simpler zig-zag channel geometries with a single channel (see Figure 3c top right).

As shown in Figure 3d,e, mixing was achieved through a three dimensional woven channel structure, separating and re-merging fluid elements, while changing direction in all three orthogonal directions. As observed from Figure 3e, the liquid velocity increases as the channel diameter decreases in positions where liquid streams are merged and changes direction in the z-direction. In locations of changes between x or y direction of the channel-braid (marked orange) a higher liquid exchange between the two streams is predicted by the simulation than in areas marked yellow. Multi-material 3D printing of the mixing insert enables efficient reciprocation-mixing at low spin speeds on single-step fabricated fluidic parts, while allowing for design freedom for the channel geometry.

### 3.3. Reproducibility of Reciprocation

**Figure 4:**
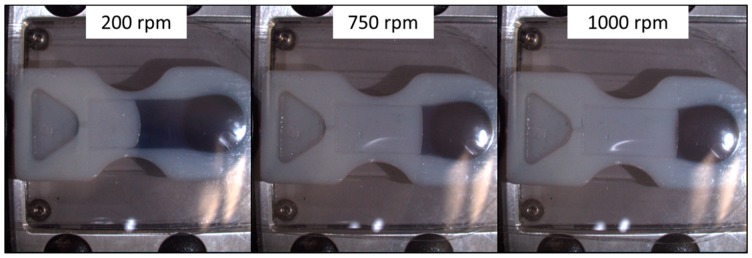
Fluid position in straight channel insert when spinning at 200 RPM, 750 RPM, and 1000 RPM respectively.

To evaluate the possible fatigue of the rubber-like material, 300 µL of dyed deionized water was loaded into the straight-channel reciprocation insert with glossy surface finish (see Figure 3b) and the insert was spun reciprocating between 400 and 1000 RPM. No change in fluid location at either spin speed was observed throughout 114 reciprocations. After 114 alternations, fluid started to leak from the insert between the adhesive and the printed part. Repeatable fluid return throughout this high number of reciprocations demonstrates that there is only minimal fatigue of the elastic membrane. Through following up with a second experiment at a continuous high spin speed we ensured that the membrane was nowhere close to catastrophic failure. During this second experiment, the same insert was resealed with a fresh top adhesive and 600 µL of DI water was loaded into the fill chamber. The insert was then spun at 2000 RPM and the liquid level was observed. In addition, in this experiment, no significant change of the liquid level could be observed throughout 14 min of continuous spinning. After 15 min, the adhesive started to delaminate and leakage from the insert was observed. We therefore conclude that the fatigue of the elastic material is not significant when used for a microfluidic application such as the one we demonstrated. Figure 4 shows the liquid positions at different spin speeds. The matte surface finish did not allow for proper bonding between the printed part and the adhesive and leakage from the insert was already observed during the first acceleration to a spin speed of 1000 RPM.

### 3.4. Surface Characterization

**Figure 5:**
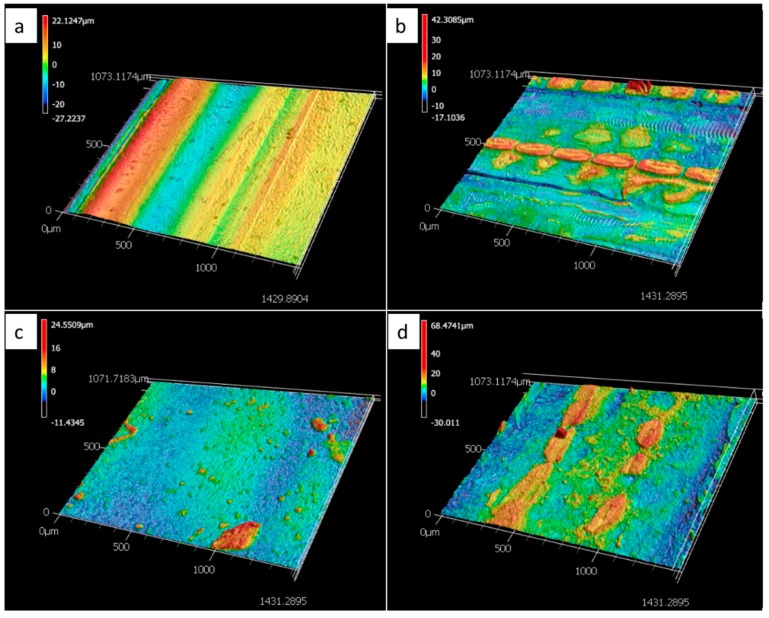
3D images of the surface topography of the printed surfaces, (**a**) VeroWhitePlus, glossy, (**b**) VeroWhitePlus, matte, (**c**) TangoBlackPlus, glossy, (**d**) TangoBlackPlus matte.

To characterize the printed surfaces for microfluidic applications, 3D surface images were taken and surface roughness was measured with a Keyence VK-X250 confocal microscope (Keyence Corp., Osaka, Japan). Furthermore, contact angles (DI water-air interface) were analyzed with a Kruess Drop Shape Analyzer DSA30 (KRÜSS GmbH, Hamburg, Germany). The 3D images of the surfaces are presented in Figure 5 and the surface roughness perpendicular to printing direction and contact angles are listed in Table 1. The surface roughness was approximately one order of magnitude higher than that obtained by Walczak et al., who analyzed the roughness of glossy and matte surfaces of parts printed on an Objet30Pro, measuring 0.19 µm and 0.61 µm respectively [18]. Udroiu et al. present average surface roughness measurements of parts obtained on an EDEN 350 Polyjet printer, yielding 1.04 µm for a matte and 0.84 µm for a glossy surface finish, respectively [19]. The reason for a higher roughness of the glossy than the matte part in our tests is likely due to the directional dependency of the surface finish. The glossy surface finish, especially, leads to grooves along the print direction. An evaluation of directional dependency is described elsewhere [18].

**Figure 6:**
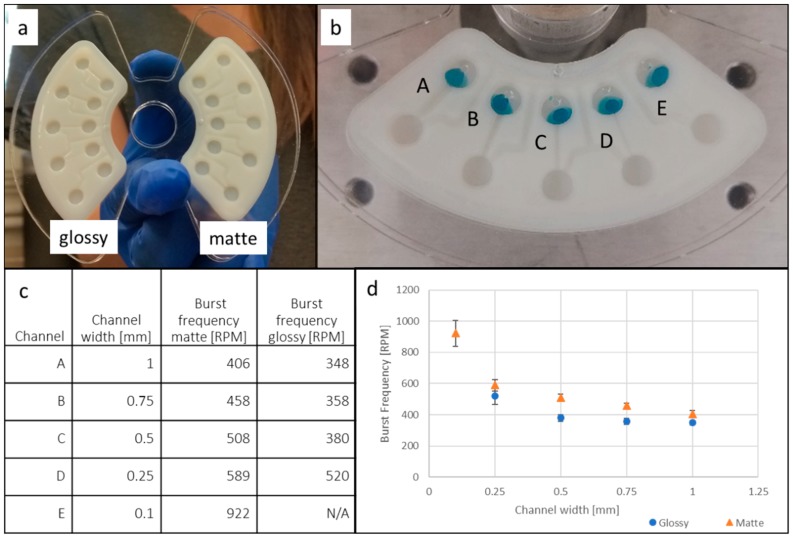
Burst frequency testing of 3D printed inserts with glossy and matte surface finish. (**a**) Inserts with different surface finish in a laser-cut acrylic holder. (**b**) Naming convention of channels, with decreasing channel widths left to right. (**c**) Average burst frequencies for channels A through E. Channel E did not resolve in the glossy surface finish part due to limited resolution. (**d**) Graph of burst frequency as a function of channel width. Blue/round symbols are burst frequencies of glossy and orange/triangular are burst frequencies for the matte part. Error bars represent the standard deviation. The number of tests for each channel was 10 and the volume of deionized dyed water per channel was 20 µL.

As can be seen from Table 1, surface contact with support material (i.e., matte surface finish) during the printing process not only affects the surface roughness but also the contact angles. The static contact angle for matte, as compared to glossy finish, is more hydrophobic, thus contributing to higher burst pressures for the same channel geometries (compare Figure 6d) [20]. The effect of the support material on the surface topography of the part can be gleaned from Figure 5. While the 3D scan of the matte surface in Figure 5b shows remaining drop-shaped material, as deposited by the printer head, the glossy finish, as shown in Figure 5a, only shows material ridges and trenches along the print direction. To further characterize the microfluidic behavior of the printed parts, burst frequency measurements were taken on the parts shown in Figure 6. The naming of channels is shown in Figure 6b and the average burst frequencies and nominal widths are shown in Figure 6c,d. Standard deviations (based on 10 tests each) are shown as error bars in Figure 6d. Channel E did not resolve in the glossy surface finish part due to limited print resolution. Besides promoting a matte surface finish, the utilization of support material yields higher dimensional accuracy and allows for smaller feature size. When using support material, liquid resin droplets expelled from the print head onto the outer layer(s) of the printed part, are held in position by support material in the adjacent layer. In parts with a glossy surface these material droplets are able to spread and fill groves and pits before being cured by UV light, thus affecting the resolution of features and affecting the topography of the surface.

Overall it can be seen from Figure 6 that the matte surface leads to higher burst frequencies than the glossy surface finish. This is likely due to a combination of inherent surface topography and hydrophobicity, thus affecting capillary forces in the channel. Furthermore it can be seen that burst frequency increases steeply at very small channel widths, as described in the burst valve theory discussed in Bauer et al. [20].

## 4. Conclusions

We have demonstrated reciprocation and mixing on a microfluidic CD using 3D printed dual material inserts. Mixing and reciprocation enable improved detection, reaching a lower LOD in a shorter amount of time. Generally, utilization of an elastic membrane in combination with an unvented air chamber allows for reciprocation at lower spin speeds, while utilizing a fluid filled chamber with an elastic membrane allows for a simpler design and a reduced footprint. Mixing was achieved through reciprocating flow through a 3D woven channel and the simulation showed the locations of the highest mixing efficiency within the channel.

While 3D printing is only appropriate for prototyping of CD inserts rather than mass manufacturing due to its sequential nature of producing parts, material jetting of a flexible and a rigid material can be used to model products before investing in a costly mold. Printed surfaces were fluidically evaluated as differences in surface properties need to be considered when utilizing a prototyping approach to correctly predict fluidic behavior of manufactured parts [20]. The surface finish of the printed parts not only impacts hydrophilicity and topography but also the smallest possible dimensions with matte surface finish, allowing for more accurate features, while the glossy surface finish yields higher hydrophilicity and better bonding with adhesives.

## Figures and Tables

**Table 1:** Surface roughness and contact angles of printed materials.

Surface	Average Surface Roughness Ra (μm)	Average Static Contact Angle (°)
VeroWhitePlus Glossy	5.6	94.4
VeroWhitePlus Matte	3.3	105.3
TangoBlackPlus Glossy	1.2	97.5
